# Clinicians’ preferences and attitudes towards the use of lithium in the maintenance treatment of bipolar disorders around the world: a survey from the ISBD Lithium task force

**DOI:** 10.1186/s40345-023-00301-y

**Published:** 2023-05-27

**Authors:** Diego Hidalgo-Mazzei, Tim Mantingh, Xavier Pérez de Mendiola, Ludovic Samalin, Juan Undurraga, Sergio Strejilevich, Emanuel Severus, Michael Bauer, Ana González-Pinto, Willem A. Nolen, Allan H. Young, Eduard Vieta

**Affiliations:** 1 Bipolar and depressive disorders unit, Department of Psychiatry and Psychology, Hospital Clínic, IDIBAPS, University of Barcelona, 170 Villarroel St, 08036 Barcelona, Spain; 2 Departament de Medicina, Facultat de Medicina i Ciències de la Salut, Universitat de Barcelona (UB), C. Casanova, 143, 08036 Barcelona, Spain; 3 Centro de Investigación Biomédica en Red de Salud Mental (CIBERSAM), Instituto de Salud Carlos III, Madrid, Spain; 4 Department of Psychological Medicine, Institute of Psychiatry, Psychology and Neuroscience , King’s College London, London, UK; 5 Bioaraba, Psychiatry Service, Department of Neurosciences, Research Group on Severe Mental Illness, Araba University Hospital, University of the Basque Country UPV/EHU, Osakidetza, Vitoria-Gasteiz, Spain; 6 Department of Psychiatry, CHU Clermont-Ferrand, University of Clermont Auvergne, CNRS, Clermont Auvergne INP, Institut Pascal (UMR 6602), Clermont-Ferrand, France; 7 Department of Neurology and Psychiatry, Clinica Alemana Universidad Del Desarrollo, Santiago, Chile; 8 Área, Asistencia e investigación en trastornos del ánimo, Buenos Aires, Argentina; 9 Department of Psychiatry and Psychotherapy, University Hospital Carl Gustav Carus, TU Dresden, Dresden, Germany; 10 Department of Psychiatry, University Medical Center Groningen, University of Groningen, Groningen, The Netherlands

**Keywords:** Lithium, Bipolar disorders, Treatment, Maintenance, Survey, Use, Preferences, Attitudes

## Abstract

**Background:**

Lithium has long been considered the gold-standard pharmacological treatment for the maintenance treatment of bipolar disorders (BD) which is supported by a wide body of evidence. Prior research has shown a steady decline in lithium prescriptions during the last two decades. We aim to identify potential factors explaining this decline across the world with an anonymous worldwide survey developed by the International Society for Bipolar Disorders (ISBD) Task Force “Role of Lithium in Bipolar Disorders” and distributed by diverse academic and professional international channels.

**Results:**

A total of 886 responses were received of which 606 completed the entire questionnaire while 206 completed it partially. Respondents were from 43 different countries comprising all continents. Lithium was the most preferred treatment option for the maintenance of BD patients (59%). The most relevant clinical circumstances in which lithium was the preferred option were in patients with BD I (53%), a family history of response (18%), and a prior response during acute treatment (17%). In contrast, Lithium was not the preferred option in case of patients´ negative beliefs and/or attitudes towards lithium (13%), acute side-effects or tolerability problems (10%) and intoxication risk (8%). Clinicians were less likely to prefer lithium as a first option in BD maintenance phase when practising in developing economy countries [X2 (1, N = 430) = 9465, p = 0.002) ] and private sectors [X2 (1, N = 434) = 8191, p = 0.004)].

**Conclusions:**

Clinicians’ preferences and attitudes towards the use of lithium in the maintenance treatment of bipolar disorders appear to be affected by both the patients’ beliefs and the professional contexts where clinicians provide their services. More research involving patients is needed for identifying their attitudes toward lithium and factors affecting its use, particularly in developing economies.

**Supplementary Information:**

The online version contains supplementary material available at 10.1186/s40345-023-00301-y.

## Background

Lithium has long been considered a key and gold-standard pharmacological treatment for mood disorders. Its cost-effectiveness in the long-term treatment of mood disorders, and especially in bipolar disorders (BD), has been supported by numerous randomized clinical trials, observational studies and meta-analyses (Burgess et al. 2001; Smith and Cipriani 2017). Its use as a first-line treatment option during maintenance phase of BD is widely supported by most international guidelines (Kessing 2019a; Malhi et al. 2017). A renewed interest in the academic field has also highlighted lithium as the only pharmacological compound in mood disorders with antisuicidal properties in recent decades (Smith and Cipriani 2017). More recently, even potential neuroprotective as well as antiviral effects were proposed when used in lower doses than those recommended in BD maintenance treatment (i.e., 0.60–0.80 mmol/L) (Dell’Osso et al. 2016; Murru et al. 2020; Nolen et al. 2019; Won and Kim 2017).

Despite lithium’s obvious advantages and the long experience of clinicians prescribing it, it is generally assumed it requires more initial and regular assessments and tests (i.e. ECG, blood plasma levels, renal and thyroid-parathyroid function tests) compared to other mood stabilizers (Nolen et al. 2019). In addition, there is still conflicting evidence regarding the long-term effects of its use on the kidneys (Nielsen et al. 2018; Schoretsanitis et al. 2022). Nevertheless, it is not clear whether the aforementioned reasons have contributed to the steady decline in lithium prescriptions in mood disorders in several countries and regions during the last two decades (Karanti et al. 2016; Rhee et al. 2020; Young and Hammond 2007). Given its many benefits in comparison to its inconveniences, this reduction in the use of lithium is especially worrisome considering the current lack of better pharmacological with similar properties in BD (Fountoulakis et al. 2022; Nestsiarovich et al. 2022; Young and Hammond 2007).

Considering this context, the Lithium Task Force (TF) of the International Society of Bipolar Disorders (ISBD) in collaboration with the International Group for the Study of Lithium-Treated Patients (IGSLI)-has launched a series of initiatives to explore the reasons and potential problems that could be influencing the prescription of lithium around the world while also work on recommendations about its appropriate use and monitoring (Grillault Laroche et al. 2020; Nolen et al. 2019; Shulman et al. 2019). Among these initiatives, it was decided that an anonymous worldwide survey collecting clinicians’ lithium prescription patterns and preferences could provide useful data regarding potential obstacles concerning the use of lithium in the maintenance treatment of bipolar disorders according to its scientific evidence. Identifying factors will allow the TF to plan more specific initiatives and actions to address potential issues influencing lithium prescription worldwide.

## Methodology

In order to accomplish our aim, a first set of initial questions based on theliterature and previous studies (Pérez de Mendiola et al. 2021; Strejilevich et al. 2011) were outlined (DHM, TM, AY). This first draft was distributed to international members of the TF (WN, MB, ES,AG, EV) to incorporate all potential local and general preferences, issues, alongside limitations in Lithium prescription. After three iterative rounds, a final version of the questionnaire with 29 items was agreed upon (Additional file 1), which was subsequently formatted and uploaded to the Hospital Clínic of Barcelona survey platform (https://enquesta.clinic.cat/). Following a one-week internal technical and consistency check, the link to the survey was distributed among the mailing list of the ISBD and IGSLI members as well as several other professional organizations around the world involved in the care of people with BD and further distributed by diverse academic and professional international channels. The mail also requested colleagues and members to re-distribute the invitation within their local institutions as well as regional and national professional associations. The link to the survey was also announced in strictly closed professional groups on social networks (i.e., Facebook and LinkedIn). After accessing the survey, a protective CAPTCHA challenge-response test was set to prevent automatic responses followed by a brief introduction about the survey’s aims and specific questions used conditional logic to avoid redundant questions (e.g., If lithium was not available to prescribe in the country where the clinician provided services, further questions about lithium prescription were omitted). Data collected was stored in encrypted servers only accessible to the researchers at Hospital Clínic of Barcelona. No direct (e.g. name, ID, date or place of birth, exact age, etc.) or indirect (e.g. IP address, cookies tracking) personally identifiable information was collected by the website to ensure a fully anonymous survey.

Initial access to the survey was planned for 1 year, starting in August 2020. However, an additional 3-month extension to November 2021 was agreed upon among the TF due to the circumstances arising from the COVID-19 pandemic during the survey distribution period.

We conducted descriptive analyses to depict the respondents’ sociodemographic and professional characteristics. As no mandatory response to any specific question was requested, this resulted in a variable number of responses for each item. Hence, each question was analyzed independently to characterize the sample. Within groups´ percentages were computed to make associations and inferences between variables of interest. Chi-square tests were conducted to determine specific differences (i.e., sex, age, years practising, sector, context and country providing services) of those respondents preferring to use lithium as a first treatment option. Countries´ economic category were determined by the last edition (April 2022) of International Monetary Fund (IMF) (International Monetary Fund IMF 2022). Lithium monitoring standards were adopted from the last recommendations of the ISBD/IGSLI Lithium Task Force (Nolen et al. 2019). Statistical significance was established at *p* < .05. The data collected were analyzed using SPSS version 28 (SPSS Inc., Chicago, IL). For reporting purposes, we rounded up percentages if the next decimal was six or above, and down if it was five or less.

## Results

A total of 886 responses were received of which 606 completed the whole questionnaire while 206 completed it partially (below 50% of all questions). There were no mandatory questions, but surveys completing less than 20% of the questions were omitted from the analyses for comparative reasons (N = 68). Respondents were from 43 different countries from 5 continents, with most responses received from Argentina (14%), France (12%), Netherlands (11%), Italy (10%), Germany (9%), Spain (8%), United States (6%), Chile (5%), Brazil (4%), Canada (3%), Australia (2%), United Kingdom (2%), Mexico (1%) and Denmark (1%). Thirty per cent of the responses corresponded to clinicians from 20 different developing economies with most respondents from SouthAmerica (35%), Asia (30%) and Africa (15%). Seventy-five per cent of the clinicians were not affiliated with major mood disorders international societies distributing directly or indirectly the survey [i.e., ISBD, IGSLI, International Society for Affective Disorders (ISAD)].

The majority of the respondents were male (55%) with a large predominance of psychiatrists among them (83%) while 11% were trainees and 5% were general practitioners. There was an equivalent distribution of age range with most respondents between 25 and 35 (25%), 36–45 (26%), 46–55 (21%) and 56–65 (18%) years old. Most of them had between 6 and 15 years of practice (30%) followed by those between 16 and 34 years (28%). The setting where they provided their services was mostly public (72%) and to a lesser extent the private sector (27%). 37% of the clinicians reported having between 11 and 25% of bipolar patients in their caseload while 23% had between 26% and 50% and twenty% less than 10%. Other sociodemographic and professional characteristics are detailed in Table 1.

**Table 1:** Clinicians' sociodemographic and professional characteristics

Variables categories	N.	Percentage (%)^b^
Age		
25–35	181	25
36–45	190	26
46–55	151	21
56–64	134	18
≥ 65	75	10
Sex		
Female	324	45
Male	404	56
Professional category		
Other	35	5
Psychiatrist	610	84
Trainee/resident	83	11
Years practicing since finished training		
≤ 5	149	21
6–15	219	30
16–34	205	28
≥ 35	83	12
Trainee/resident	68	9
Sector^a^		
Public	528	73
Private	200	28
Area^a^		
Urban	545	75
Rural	28	4
Both	152	21
Level of complexity of health care centre^a^		
Primary	38	5
Secondary	338	47
Tertiary	209	29
Other	14	2
More than one	126	17
Setting^a^		
Inpatient	114	16
Outpatient	346	48
Partial hospitalization	31	4
Other	7	1
More than one setting	227	31
Patients’ age^a^		
Children (up to 12 years)	3	0
Adolescents (13–17 years)	27	4
Adults (18–65 years)	675	93
Elderly (> 65 years)	23	3

^a^Questions referred to the predominant option where clinicians’ provided their professional services

^b^Percentages are calculated within each variable

Regarding the prescription of lithium, half of the respondents reported using lithium in more than 50% of their bipolar patients as maintenance treatment while 16% to over 75% of them. 44% prescribe it with a frequency of administration of twice a day for immediate-release and 57% with a frequency of once per day for extended-release formulas.

**Fig. 1:**
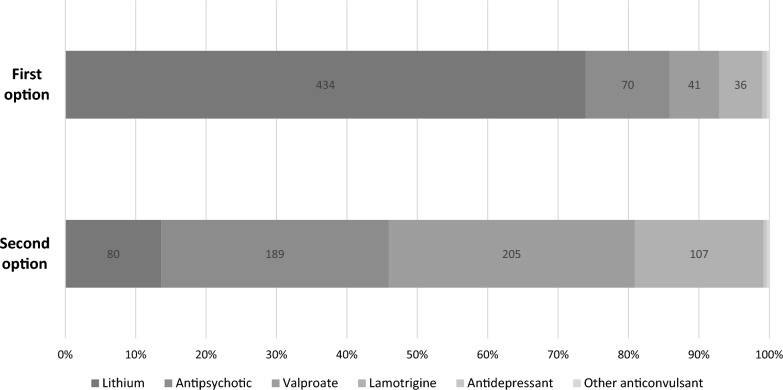
First and second choice of pharmacological treatment in the maintenance phase of BD. The bar chart shows the percentage distribution of first and second choice of pharmacological treatments in the maintenance phase of BD according to participants´ responses. In each bar section, actual number of responses for each option are displayed

Lithium salts were the most preferred first treatment option for the maintenance treatment of BD patients (74%), followed by antipsychotics (12%) and valproate (7%). Figure 1 shows first and second treatment choice percentage distributions. Table 2 details group percentage differences among variables of interest in which lithium was the preferred first option. Most of the respondents started lithium right afterthe first manic or hypomanic episode (46%) with a lesser extent prescribing it after the second episode regardless of if it is manic or depressive (13%). The 5 most relevant clinical circumstances in which lithiumwas the preferred option during the maintenance phase were in patients with bipolar I disorder (53%), a family history of response to lithium (18%), a response to lithium during the acute treatment (17%), there were current or previous suicidal thoughts or attempts (15%) and when there was a specific predominant polarity (9%).

**Table 2:** Professional characteristics of clinicians’ who preferred lithium as a first option for the maintenance treatment of bipolar disorder

Variables categories	N.	Percentage^b^
Professional category		
Psychiatrist	363	84
Trainee/resident	56	13
Other	15	4
Years practicing since finished training		
Trainee / resident	41	10
≤ 5	91	21
6–15	125	29
16–34	126	29
≥ 35	49	11
Sector^a^		
Public	329	76
Private	105	24
Area*		
Urban	326	76
Rural	14	3
Both	92	21
Country economy		
Developed	319	74
Developing	111	26
Level of complexity^a^		
Primary	10	2
Secondary	189	44
Tertiary	155	36
Other	3	1
More than one	76	18
Setting^a^		
Inpatient	66	15
Outpatient	210	49
Partial hospitalization	16	4
Other	2	1
More than one setting	139	32
Patients’ age^a^		
Children (up to 12 years)	0	0
Adolescents (13–17 years)	11	3
Adults (18–65 years)	408	94
Elderly (> 65 years)	15	4

^a^Questions referred to the predominant option where clinicians’ provided their professional services

^b^Percentages are calculated within each variable

**Fig. 2:**
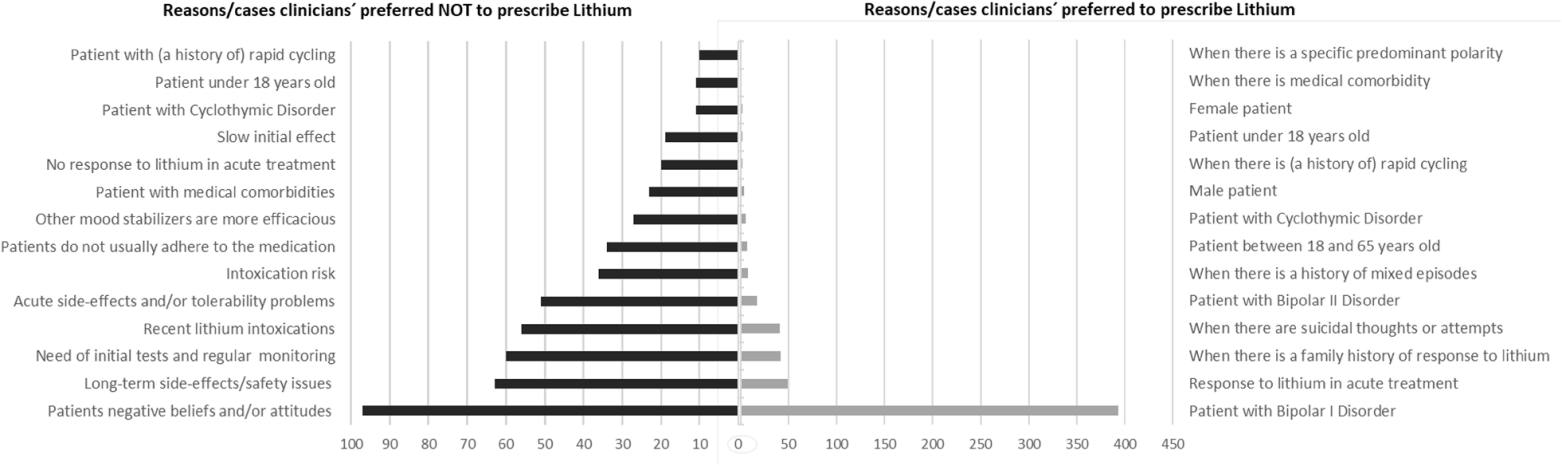
Reasons for prescribing or not prescribing Lithium for the maintenance treatment of bipolar disorder. The figure demonstrates the number of responses given for each reason/case in which participants preferred not to prescribe (left) or prescribe lithium (right)

In contrast, the most common reasons in which lithium was not the preferred option were patients’ negative beliefs and/or attitudes towards lithium (13%), acute side-effects or tolerability problems (10%), intoxication risk (8%), medical comorbidities (6%) and long-term side-effects or safety issues (metabolic, thyroid or renal dysfunction) (6%). Figure 2 shows further reasons answered by respondents in which lithium was a preferred or not preferred option for the maintenance treatment of BD. In this context, professionals prescribing lithium were more concerned with the following long-term side effects: renal function alteration (55%), hypothyroidism (29%) and weight-gain (18%).

With regard to lithium levels tests and assessments during maintenance treatment, most of the respondents ordered them routinely between 2 and 4 times per year (73%), with most of them targeting plasma levels between 0.6 and 0.8 mmol/L (52%), 0.8–1 mmol/L (14.4%) or any level between 0.6 and 1.2 mmol/L (5%). The most common additional laboratory tests routinely performed in these controls alongside lithium plasma levels were renal (78%) and thyroid (78%) functions, and electrolytes (59%), with serum calcium tests requested only by 38% of the respondents. A minority also ordered an ECG (6%) and a thyroid gland ultrasound (3%). Importantly, weight and Body mass index (BMI) were assessed frequently during routine controls by 52% of the participants. Overall, 73% of the participants responded that they included all recommended tests (i.e. BMI, lithium plasma levels, electrolytes, renal and thyroid function and calcium). About half of the surveyed responded that they followed a guideline or protocol for systematically monitoring levels, but only a quarter of them used a standardized instrument to evaluate response to the treatment.

Considering variables of interest, a Chi-square test was conducted to explore whether professionals’ characteristics were related to the preference to use lithium as the first option in the maintenance treatment of BD. Two variables of interest showed a statistically significant relationship between those preferring lithium as a first option: clinicians were less likely to prefer lithium as a first option in BD maintenance phase when practising in developing economy countries [X2 (1, N = 430) = 9465, p = 0.002) and private sectors (X2 (1, N = 434) = 8191, p = 0.004)]. Considering these differences, we further conducted analyses to explore if differences in these variables were also translated to recommended lithium monitoring frequency (i.e. 2 or more per year) and lithium plasma levels (i.e. 0.4–1 mmol/L) during maintenance phases. No statistically significant differences were found in these parameters between developing economies or practising in the private sector.

## Discussion

With this survey, we aimed to capture attitudes toward the use of lithium in the maintenance treatment of BD, and we involved a significant sample of diverse mental health professionals from different regions around the world. Respondents´ personal and professional characteristics reflected in the survey results confirm this diversity. This allows us to extract some generalizable conclusions while also identifying some specific patterns influencing the preference to prescribe lithium upon which actions can be taken.

In general, our results indicating that clinicians use lithium in more than 50% of their BD patients as maintenance treatment are in line with previous reports and registries from diverse countries (Lin et al. 2022; Mandal et al. 2019; Pacciardi et al. 2017; Pérez de Mendiola et al. 2021; Sköld et al. 2021). However, there is a striking diversity of prescriptions patterns among different countries (Kessing 2019b), with some countries and regions having lithium prescription patterns below 50% (Grover et al. 2021; Heeren et al. 2011; Karanti et al. 2016; Lyall et al. 2019) or above (Bohlken et al. 2020; Pérez de Mendiola et al. 2021). Based on our results and due to the lack of similar precedent worldwide survey, it is difficult to confirm or not a general decline in lithium prescriptions as it has been previously reported by studies based on data from national registries. However, recently Reed et al. analyzed 20-yeard prescriptions trends in the treatment of BD from systematic national surveys to clinicians in United Stated and found a decrease in use of lithium from 30.4% to 1997 to 17.6% in 2016 (Rhee et al. 2020). The much higher percentage of lithium prescription in our sample in comparison to the results by Rhee et al. might be explained by the specific regional and health system particularities of where the survey was conducted (i.e., United States) as well as the systematic random sampling of the surveyed clinicians in comparison to our anonymous convenience sample from professional associations. Nonetheless, it can be inferred from our results that clinicians around the world have a common preference, training, and monitoring standards for lithium, including trainees, but external factors might play a role in their decision to prescribe it.

Despite the participants’ heterogeneity, it can be assumed from the general preferences and attitudes reported in the survey, that lithium use and monitoring levels generally adhere to international guidelines. Though it is one of the oldest psychopharmacological compounds, the lack of a uniform consensus among health professionals regarding its use and monitoring is still concerning (Malhi et al. 2017). Disparities in the most appropriate maintenance plasma levels and monitoring frequencies, as well as needed tests, have been continuously reported within institutions and countries around the world (Janet A Butler 2009; Nederlof et al. 2018; Nikolova et al. 2018; Paton et al. 2010; Sköld et al. 2021).

The lower preference for lithium among developing nations and the private sector is noteworthy. In the case of lithium underutilization in developing countries, there is a lack of previous studies to compare with. Moreover, there in our sample there is imbalance between developing and developed economies in which the former has significant underrepresented regions (i.e. Caribbean, Central America). Even though clinicians’ false beliefs, misconceptions and lack of training might play a role, it is also reasonable to suggest that the lack of resources allowing appropriate monitoring makes lithium a less attractive choice in comparison to those not requiring periodic monitoring and testing. Other factors that may play a role are geographical and transportation barriers to access periodic controls due to unequal distribution of health care centers and sociocultural and religious beliefs and stigma held by local communities (Muhorakeye and Biracyaza 2021; Rathod et al. 2017). Aside from the lack of infrastructure, even if they are available, test costs might also play a role in the decision to prescribe lithium in countries with limited universal healthcare (Andrade et al. 2014). The same costs-associated reasons could explain the lower preference for lithium prescription among mental health professionals from private sectors where patients need to pay the price of required tests out-of-pocket. In both cases, the lack of systematic registries and data about the actual use of lithium represents a barrier difficult to overcome in order to extract firm conclusions (Williams and Boren 2008). Nonetheless, it must be emphasized that despite the involved test costs in the use of lithium its final cost-effectiveness in both developed and developing countries is still superior to valproic acid and antipsychotics (Chisholm et al. 2005). In addition, the mhGAP Intervention Guide of the World Health Organization (WHO) reinforces this recommendation (World Health Organization, WHO 2016).

In contrast with previous surveys, we found that the most common reason for not prescribing lithium was based on the patient’s perspective, instead of the mental health care professional’s concerns about side effects. These results may reflect a growing number of negative beliefs or attitudes about lithium salts in the general population and the rapid dissemination of the misconceptions through social media (Kessing 2019a; Malhi et al. 2020). This is not a specific issue about either lithium or psychiatry as a discipline, but a new extended problem throughout the health care sector (Chou et al. 2018; Khullar 2022). In the case of lithium, these misconceptions might be also prompted in part by false beliefs about the availability of more modern, effective, and tolerable compounds such as second-generation antipsychotics (SGA) which are also approved for the maintenance of BD (Jauhar and Young 2019; Malhi et al. 2020). SGAs are generally supported by a robust marketing plan from pharmaceutical companies (Malhi et al. 2009; Young and Hammond 2007). However, no SGA has performed as well as lithium in so many crucial long-term prognosis factors of BD as lithium (Lindström et al. 2017; Selle et al. 2014). While SGAs do not require so much baseline or therapeutic monitoring, metabolic syndrome, drowsiness, sexual dysfunction, and extrapyramidal effects are much more frequent in comparison to lithium (Miura et al. 2014).

Hence, our results stress the increasing need for mental health institutions and scientific societies to counter these false beliefs with more educational and promotional campaigns about lithium targeting the general population (Bauer 2022; Chou et al. 2018; Rybakowski 2022). Likewise, closer collaboration with patients´ associations could help further explore and understand their perspectives and beliefs about lithium (Gomes et al. 2022; Jørgensen and Rendtorff 2018). The move toward a more shared-decision-making (SDM) psychiatry could also represent another interesting approach to mitigate this belief as it allows time and space to build a structuredand informed decision-making process where doubts, fears and facts can be openly discussed between patients and clinicians. It has been proposed an SDM approach can improve patient satisfaction and medication adherence in recurrent mood disorders (Samalin et al. 2018a) with several studies still ongoing to confirm this hypothesis in BD (Samalin et al. 2018b).

This research has several limitations that must be taken into account to interpret these results. First, as in all anonymous survey-based studies, the information provided by respondents is a subjective, partial and non-quantitative perspective of the topics explored. Anonymous responses make it possible to obtain more honest answers without respondents feeling judged or tested. However, most of the results at least concerning lithium prescription are in line with studies using national registries. Secondly, the possibility of a sampling bias was compensated by using all possible survey distribution channels to reach colleagues around the world the TF members, including social networks. This was reflected in the diverse, although imbalanced, representation of almost all regions around the world of the respondents. However, given the limited sample size and some underrepresented regions, results cannot be generalizable across all countries. A further frequent issue is that online surveys distributed by national and international scientific associations and academic institutions do not reach sectors such as private practices. Colleagues associated with these associations are frequently more exposed to training programs, conferences and guidelines which constantly update them on the use of lithium (Pérez de Mendiola et al. 2021). Nonetheless, 75% of colleagues were not affiliated at all with major international mood disorder societies, and almost 28% provided their services in the private sector. Thus, we consider the survey distribution strategy was successful at reaching a representative sample of real-world clinicians, most of them not affiliated to the mood disorders societies. Finally, it is important to stress that as a survey primarily designed and announced with the aim of exploring the use of lithium in the maintenance of BD. Hence, clinicians who don’t see a substantial role for lithium for this indication might be less inclined to take part in the survey in comparison to those who feel strongly positively about it.

## Conclusion

Overall, this study highlights the heterogeneous patterns of lithium prescription around the world. These patterns appear to be affected by both the patients’ beliefs and the professional context and region in which the clinicians operate, despite the strength of the evidence-base in favor of lithium and its safety (Carvalho et al. 2021; Gomes-da-Costa et al. 2022). More research involving patients is needed for identifying their attitudes toward lithium. There is also a need for more objective data in developing economies to determine and sort out factors influencing the use of lithium.

## Supplementary Information

**Additional file 1.** The supplementary file contains the complete survey answered by the participantsof the study about psychiatrists’ personal concepts, opinions and experiences on the clinical use of lithium in the maintenance treatment of bipolar disorders. For each survey section, the question, instructions to respond, and count of the responses to each possible answer are shown.

## Data Availability

The dataset used in this study is available upon reasonable request from researchers of academic institutions to the ISBD/IGSLI Task Force on treatment with lithium.

## Abbreviations

BD: Bipolar disorder
BMI: Body mass index
ISBD: International society of bipolar disorders
IGSLI: International group for the study of lithium-treated patients
TF: Task force
