# Supplementary material for: Clinicians’ preferences and attitudes towards the use of lithium in the maintenance treatment of bipolar disorders around the world: a survey from the ISBD Lithium task force
Source: Int J Bipolar Disord. 2023 May 27;11:20. doi: 10.1186/s40345-023-00301-y (PMC10220344; doi:10.1186/s40345-023-00301-y)
Supplement: Supplementary file 1 — Additional file 1. The supplementary file contains the complete survey answered by the participantsof the study about psychiatrists’ personal concepts, opinions and experiences on the clinical use of lithium in the maintenance treatment of bipolar disorders. For each survey section, the question, instructions to respond, and count of the responses to each possible answer are shown. [file 40345_2023_301_MOESM1_ESM.pdf]

The following survey is aimed to collect psychiatrists' personal concepts, opinions and experiences on the clinical use of lithium in maintenance treatment of bipolar disorders. Please answer each item based on your personal experience and opinions. It should take around 10 to 15 minutes to complete this questionnaire. The information collected will be analysed anonymously. Thank you very much for your time and cooperation.

Section A: Sociodemographics

Please complete the following information selecting one option for each multiple-choice item.

A1. Age:

- 25 - 35 ☐
- 36 - 45 ☐
- 46 - 55 ☐
- 56 - 64 ☐
- ≥ 65 ☐

A2. Gender:

- Female ☐
- Male ☐

Section B: Practice characteristics

Please complete the following information selecting one option for each multiple-choice item, except for those selected questions allowing multiple options which are specified below them.

B1. Profession:

- Psychiatrist ☐
- Trainee / resident ☐
- Other ☐

Other

[ ]

B2. **Years practising as a psychiatrist since finished training:**

- ≤5 ☐
- 6 - 15 ☐
- 16 - 34 ☐
- ≥ 35 ☐
- Trainee / resident ☐

B3. **Context in which you predominantly provide your services:**

- Public ☐
- Private ☐

B4. **Area in which you predominantly provide your services**

- Urban ☐
- Rural ☐
- Both ☐

B5. Country where you usually provide your services:

- Afghanistan ☐
- Albania ☐
- Algeria ☐
- Andorra ☐
- Angola ☐
- Antigua & Deps ☐
- Argentina ☐
- Armenia ☐
- Australia ☐
- Austria ☐
- Azerbaijan ☐
- Bahamas ☐
- Bahrain ☐
- Bangladesh ☐
- Barbados ☐
- Belarus ☐
- Belgium ☐
- Belize ☐
- Benin ☐
- Bhutan ☐
- Bolivia ☐
- Bosnia Herzegovina ☐
- Botswana ☐
- Brazil ☐
- Brunei ☐
- Bulgaria ☐
- Burkina ☐
- Burundi ☐
- Cambodia ☐
- Cameroon ☐
- Canada ☐

B6. Level of healthcare service in which you predominantly provide your services:

- Primary care (e.g. general practitioner, community service) ☐
- Secondary care (e.g. hospitals/specialists) ☐
- Tertiary care (e.g. specialized affective / bipolar disorders services) ☐
- Other, namely: ☐

Other, namely:

B7. Setting in which you predominantly provide your services:

- Inpatient ☐
- Outpatient ☐
- Partial hospitalisation (e.g. Day Hospital, Home-treatment) ☐
- Other, namely: ☐

Other, namely:

B8. To which age group do most of your patients belong?

- Children (up to 12 years) ☐
- Adolescents (13-17 years) ☐
- Adults (18-65 years) ☐
- Elderly (>65 years) ☐

B9. Are you an active member of one or more of the following organisations?

- International Society for Bipolar Disorders (ISBD) ☐
- International Society for Affective Disorders (ISAD) ☐
- International Group for the Study of Lithium Treated Patients (IGSLI) ☐
- None of the above ☐

Clínic Barcelona | UNIVERSITAT DE BARCELONA

B10. **What is the approximate number of patients under your care in a given year (clinical caseload)?**

- Less than 25 ☐
- Between 26 and 75 ☐
- Between 76 and 150 ☐
- More than 150 ☐

B11. **What percentage of those patients have a diagnosis of bipolar disorders?**

- Less than 10% ☐
- Between 11 and 25% ☐
- Between 26 and 50% ☐
- Between 51 and 75% ☐
- More than 76% ☐

Section C: Lithium prescription

Please complete the following information selecting one option for each multiple-choice item, except for those selected questions allowing multiple options which are specified below them.

C1. **Is Lithium as a pharmacological treatment available to prescribe in your country?**

- Yes ☐
- No ☐

C2. **To what percentage of your patients with bipolar disorders do you estimate you are currently prescribing lithium salts for maintenance treatment?**

- ≤ 5 % ☐
- 6 - 25 % ☐
- 26 - 50 % ☐
- 51 - 75 % ☐
- 76 - 100 % ☐
- I don´t prescribe lithium at all ☐

C3. Please rank among the following statements the 5 most important reasons/cases you prefer NOT TO PRESCRIBE Lithium for maintenance treatment of bipolar disorders patients?

Other mood stabilizers (anticonvulsants and/or antipsychotics and/or other) are more efficacious	
Slow initial effect	
Need of initial tests and regular blood levels monitoring	
Patients negative beliefs and/or attitudes about lithium	
High risk of relapse/recurrence after discontinuation	
Acute side-effects and/or tolerability problems	
Long-term side-effects/safety issues (metabolic and/or thyroid and/or renal)	
Intoxication risk	
Interaction with other pharmacological treatments	
Didn´t receive enough training to prescribe it with confidence	
Inconsistent availability/cost at local pharmacies	
No response to lithium in acute treatment	
Patient with Bipolar I Disorder	
Patient with Bipolar II Disorder	
Patient with Cyclothymic Disorder	
Patient under 18 years old	
Patient between 18 and 65 years old	
Patient over 65 years old	
Patient with suicidal thoughts or attempts	
Female patient	
Male patient	
Patient with (a history of) rapid cycling	
Patient with medical comorbidities	
Patient with psychiatric comorbidities	
Patient with a history of mixed episodes/episodes with mixed features	
Patients do not usually adhere to the medication	
Many Lithium intoxications in the last few years/months	
Cognitive problems	

C4. **Please rank among the following statements the 5 most important clinical circumstances you DO PREFER TO PRESCRIBE Lithium for maintenance treatment of bipolar disorders patients?**

Patient with Bipolar I Disorder ☐☐

Patient with Bipolar II Disorder ☐☐

Patient with Cyclothymic Disorder ☐☐

Patient under 18 years old ☐☐

Patient between 18 and 65 years old ☐☐

Patient over 65 years old ☐☐

Female patient ☐☐

Male patient ☐☐

Response to lithium in acute treatment ☐☐

When there is a family history of response to lithium ☐☐

When there are suicidal thoughts or attempts ☐☐

When there is psychiatric comorbidity ☐☐

When there is medical comorbidity ☐☐

When there is (a history of) rapid cycling ☐☐

When there is a specific predominant polarity ☐☐

When there is a history of mixed episodes /episodes with mixed features ☐☐

C5. **Please rank among the following long-term side-effects the 3 of them you are most concerned about when you prescribe lithium?**

Renal function alteration ☐

Hypothyroidism ☐

Hyperparathyroidism ☐

Metabolic (weight gain) ☐

Cardiac arrhythmias ☐

Cognitive effects ☐

C6. Which treatment do you usually prefer as FIRST option for the maintenance treatment of patients with bipolar disorders?

- [ ] Antidepressant
- [ ] Antipsychotic
- [ ] Lithium
- [ ] Lamotrigine
- [ ] Valproate
- [ ] Other anticonvulsant
- [ ] Other, please specify

Other, please specify

C7. Which treatment do you usually prefer as SECOND option for the maintenance treatment of patients with bipolar disorders?

- [ ] Antidepressant
- [ ] Antipsychotic
- [ ] Lithium
- [ ] Lamotrigine
- [ ] Valproate
- [ ] Other anticonvulsant
- [ ] Other, please specify

Other, please specify

C8. When you prescribe lithium for the maintenance treatment of bipolar disorders, at which point of the course of the illness do you usually do it?

- After the first manic/hypomanic episode ☐
- After the second episode regardless if it is manic or depressive ☐
- After the third episode regardless if it is manic or depressive ☐
- During the first five years of the illness onset ☐
- Between 6 to 10 years after the illness onset ☐
- After 11 or more years after the illness onset ☐
- Whenever there is a lack of tolerability (adverse effects) and/or response to other options ☐
- Other, please specify ☐

Other, please specify

C9. Which range of lithium levels do you use more frequently for the maintenance treatment of bipolar disorders?

- Between 0.4 - 0.6 mmol/L ☐
- Between 0.6 - 0.8 mmol/L ☐
- Between 0.8 - 1.0 mmol/L ☐
- Between 1.0 - 1.2 mmol/L ☐
- Any level between 0.6 - 1.2 mmol/L ☐
- Other, please specify ☐

Other, please specify

C10. How many times a year do you usually monitor lithium levels in a patient with bipolar disorders with maintenance lithium treatment who didn´t require dosage adjustments or experienced symptoms (stabilized regimen)?

- None ☐
- Once/year ☐
- Twice/year ☐
- Three times/year ☐
- Four or more times/year ☐
- Other, please specify ☐

Other, please specify

C11. Which of the following additional tests/measures does your routine lithium monitoring levels include?

- Weight / BMI ☐
- Complete Blood Count ☐
- Electrolytes ☐
- Renal function tests ☐
- Serum Calcium ☐
- Thyroid function tests (blood) ☐
- Ultrasound of thyroid gland (ultrasonography) ☐
- Other, please specify ☐

Other, please specify

C12. **When you prescribe lithium immediate-release, how many times a day do you usually prescribe it?**

- Once/day in the morning ☐
- Once/day in the evening-night ☐
- Twice/day ☐
- Three times/day ☐

C13. **When you prescribe lithium extended-release, how many times a day do you usually prescribe it?**

- Once/day in the morning ☐
- Once/day in the evening-night ☐
- Twice/day ☐
- Three times/day ☐

C14. **Do you/ your centre use clinical guideline(s) for lithium prescription?**

- Yes ☐
- No ☐

C15. **Do you/your centre use clinical guideline(s) or protocol(s) for systematic monitoring of lithium levels and adverse effects?**

- Yes ☐
- No ☐

C16. **Do you/your centre use a systematic and standardized instrument (e.g. rating scale, life chart) or tool (e.g. electronic, smartphone) to evaluate the response to lithium?**

- Yes ☐
- No ☐

Your answers have been saved. Thank you for your collaboration.
